# Virtual Reality as a Surgical Care Package for Patients Undergoing Weight Loss Surgery: A Narrative Review of the Impact of an Emerging Technology

**DOI:** 10.7759/cureus.29608

**Published:** 2022-09-26

**Authors:** Ahmed Gendia, Ayman Zyada, Muhammad Talal Nasir, Mohannad Elfar, Mohamed Sakr, Masood U Rehman, Alwyn Cota, James Clark

**Affiliations:** 1 Bariatric Surgery, Royal Cornwall Hospitals NHS Trust, Truro, GBR; 2 Vascular Surgery, Northampton General Hospital, Northampton, GBR; 3 General Surgery, Northampton General Hospital, Northampton, GBR

**Keywords:** weight loss surgery, weight loss, virtual reality, bariatric surgery, vr games, virtual reality simulation, virtual reality exposure therapy, virtual reality-based rehabilitation

## Abstract

While bariatric surgery is regarded as the most effective treatment for people with severe and morbid obesity, its pathway is regarded as a complex one due to the multidisciplinary approaches required from pre-surgery education until long-term management. This is essential to maintain weight loss and improve the quality of life after bariatric surgery. Although these approaches are broadened, patient education, pre-operative preparation, behavioural therapy, rehabilitation, and dietary changes are regarded as the main domains in such complex care. With the increase in technological adaptation in medical services, virtual reality (VR) has shown many benefits that can be utilized in the care of bariatric patients undergoing surgery. However, VR has not been innovated to be a multidomain care package in which bariatric patients could benefit throughout their journey from the pre-operative optimization, recovery, and long-term follow-up. This review aims to give a brief description of some of the applications of VR technology and question whether it has the potential to be considered as a virtual ecosystem to improve the bariatric patients’ experience and pathway throughout surgery and follow-up.

## Introduction and background

Obesity constitutes an important threat to national and global public health in terms of its prevalence and rising incidence, quality of life, life expectancy, and economic burden [1,2]. In severe obesity, bariatric surgery is the most effective therapeutic option to achieve long-term weight loss and improve the associated comorbidities [3]. This has made Roux-en-Y gastric bypass (RYGB), sleeve gastrectomy (SG), and adjustable gastric banding the most popular and commonly performed bariatric surgeries [4]. However, a small proportion of patients have also been reported to not reach their optimum goal for weight loss two years after the procedure and very few can fail or regain the weight. While anatomical factors can play a part, behavioural and psychosocial optimizations are regarded as equally important. This includes eating patterns, depression, nutritional factors, and exercise [5,6].

Virtual reality (VR) development and applications have gained wide recognition in medical services by providing solutions to improve patients’ outcomes. This is through patients’ education, improving mental health, and post-operative care, including pain management, physical therapy, and rehabilitation [7,8]. VR is a computer-generated simulation of a real or imagined environment. It can be immersive or non-immersive according to its ability to involve the users [9]. The former has been the focus of many medical applications due to its ability to give the user control of the reproduced environment. Immersive virtual reality (IVR) is usually delivered in a variety of ways and the most popular being head-mounted displays or simply a headset [8].

We aim to provide insight on some of these immersive applications and how they can be included to enhance the patient pathway to optimize outcomes both in the pre- and post-operative period for patients undergoing bariatric surgery.

## Review

Methods

A systematic search following the Preferred Reporting Items for Systematic Reviews and Meta-Analyses (PRISMA) research criteria was conducted from January 2015 to December 2021. PubMed was searched using the following keywords: virtual reality, patient education, anxiety and pain, physical rehabilitation, behavioural support, obesity, eating disorders, body image, and substance cessation.

**Figure 1:**
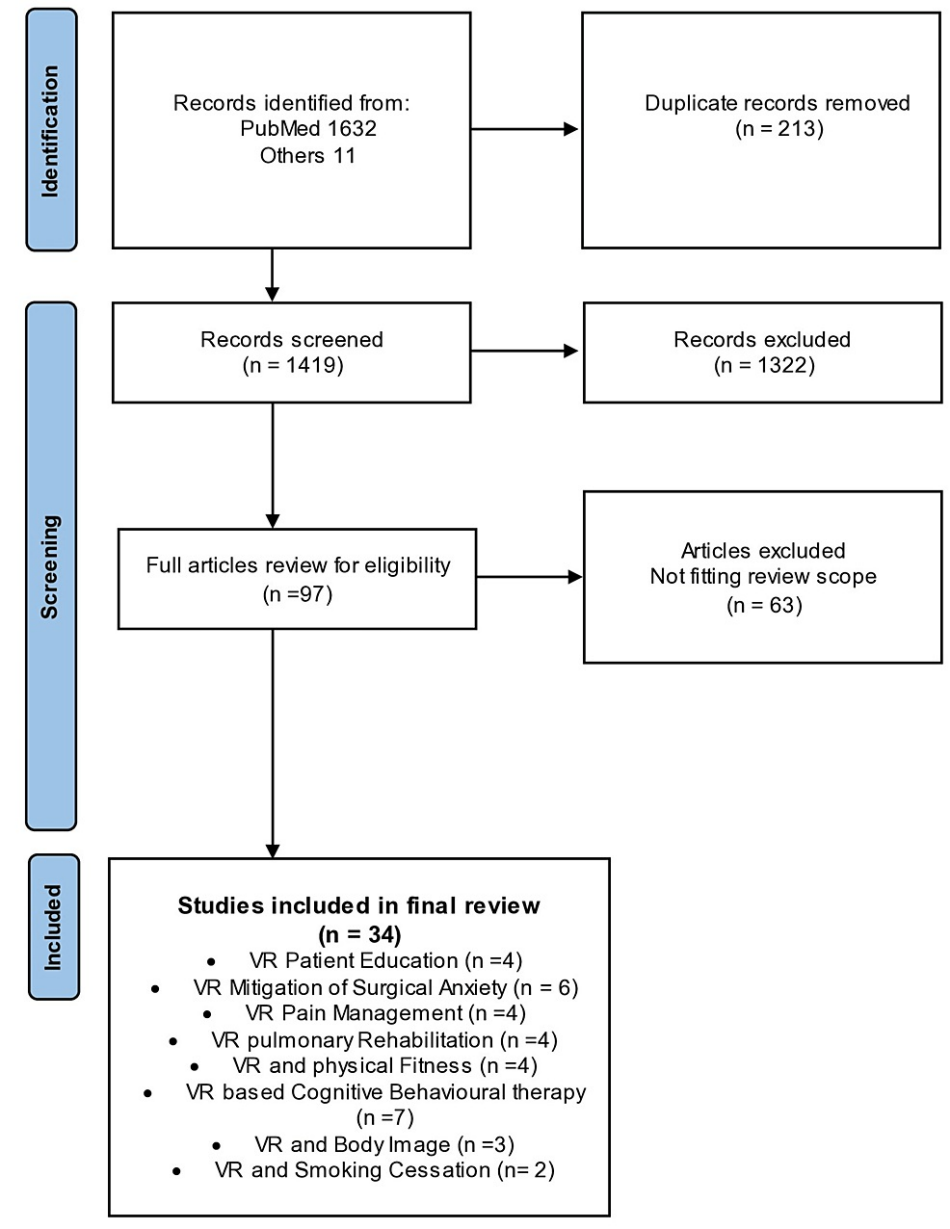
PRISMA flow chart for literature search and included studies PRISMA: Preferred Reporting Items for Systematic Reviews and Meta-Analyses; VR: virtual reality.

**Figure 2:**
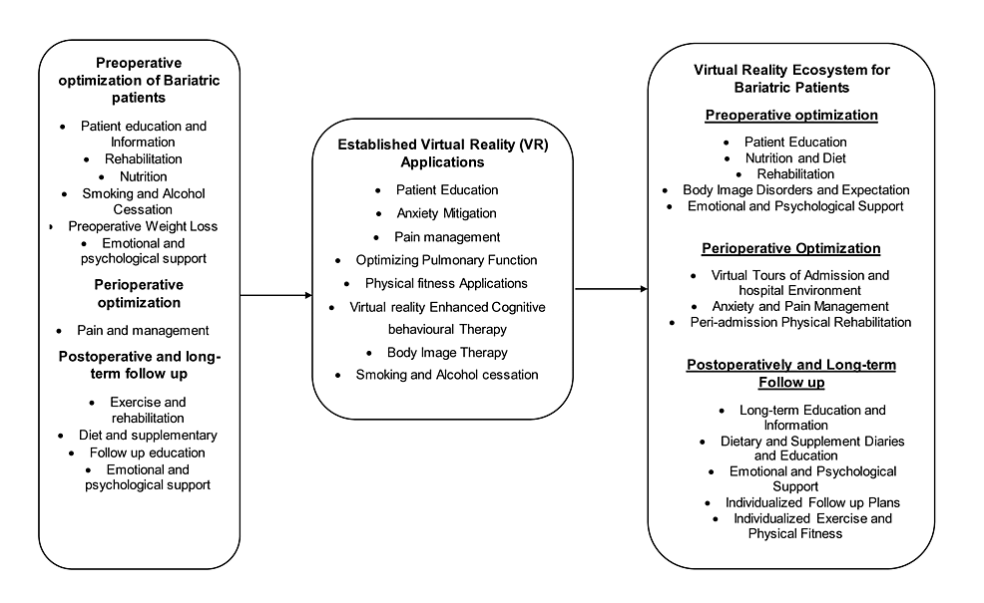
Elements of ERAS and long-term recommendations for bariatric patients and established virtual reality applications with projection into the virtual ecosystem of bariatric patients ERAS: Enhanced Recovery After Surgery.

Thirty-four studies were identified and included in the final manuscript (Figure 1) supporting VR technology across applications that can be applied to bariatric patients’ surgical pathways. The applications were subcategorized into eight different areas of interest, which can help to shape the concept of the virtual ecosystem of bariatric patients (Figure 2).

Results

VR applications have been described in the eight domains mentioned below, which can be applied in relation to patients undergoing bariatric surgery.

Virtual Reality Patient Education (VR PE)

VR education has been introduced to make the information more meaningful and patient-centred by enabling its users to be fully immersed in an interactive simulated and self-controllable visual and auditory experience [10]. In a study by Pandrangi et al. [11], VR was found to be a useful informative tool in educating patients about their aneurysmal disease through interactive reconstructed three-dimensional (3D) images of their aortic anatomy. The majority of the patients in this study agreed that VR 3D anatomy helped to improve their understanding and therefore felt more engaged in their healthcare decisions [11].

VR PE has also played a role in improving the stress levels of patients undergoing radiotherapy (RT) by improving clarity and levels of education about their treatment. A randomized study on 60 patients with chest malignancy showed that patients who received VR PE showed significant improvement in comprehension and reduction in stress and anxiety levels when compared to standard education [12]. Another study on 43 patients utilized VR PE by creating 3D images of patients in RT sessions and what to expect during the treatment. After the VR PE, 95% of patients agreed that they had a clear understanding of how they would feel when lying on the treatment table. Also, patients’ understanding of the location and the size of their cancer had significantly improved from around 50% to 95% with an increase in the orientation of side effects of the treatment by 30% post-VR PE [13].

In bariatric surgery, there is no currently reported data on the applications of VR education. However, the potential impact of VR PE can be numerous across the weight management pathway. Preoperatively, bariatric patients could potentially utilize VR to be virtually educated about different surgical options versus conservative treatment through enhanced 3D interactive images. This could be seen to help in better understanding of their options including surgery and thereby enhancing informed consent and overall education.

Post-operatively, VR-enhanced education could provide an option for daily or weekly updates on lifestyle changes, which could help in improving compliance. Importantly, this can be done from the comfort of the patient’s home with the added advantage of reducing costs and time for travelling to attend appointments.

Anxiety Related to Surgery

A significant amount of anxiety related to surgery is due to the fear and uncertainty of the outcomes. Its psychological and physical effects are associated with longer recovery, an increase in the need for analgesia, anaesthetic requirement, and unfavourable behavioural and emotional outcomes [14]. Conventional methods of mitigation of preoperative anxiety are pharmacological and non-pharmacological strategies [15].

Recently, with promising results in the management of anxiety and other psychiatric disorders, VR has been successfully applied to reduce anxiety related to surgery in different surgical settings [16]. Chan et al. [17] tested the effect of VR relaxing meditation and breathing exercises on 108 women undergoing hysteroscopy. This showed that anxiety scores were significantly reduced after the 10 minutes of VR content, which helped in reducing pain and stress related to surgery. Also, around 85% of patients reported the VR experience as good or excellent [17].

In minimally invasive abdominal surgery, Haisley et al. [18] used VR meditation as a perioperative tool with favourable results in reducing pain, anxiety, and nausea and around 75% of patients stated that they would use the VR again [18]. Similarly, VR meditation showed favourable results in reducing pain and anxiety in burns and complex pain [19,20].

The rationale for using VR to improve anxiety preoperatively is by immersing patients in a fully simulated relaxing environment with the objective of placing them in a more empowered state to deal with the triggers of their anxiety [21]. This could be applied to the bariatric population before surgery. It is to be seen from future studies whether these expected results can be validated in bariatric patients. There is therefore the potential for obtaining better evidence for patient satisfaction and reducing stress related to bariatric surgery.

Pain Management

Successful pain management is a key element of the post-operative course as it shortens recovery and reduces risks of cardiovascular and pulmonary complications. In bariatric surgery, pain management is essential to enhance recovery and prompt early mobilization, which helps to decrease venous thromboembolism, prevent other events, and reduce hospitalization [22]. Therefore, a multimodal approach through regional and systematic analgesia is considered the most effective method as it minimizes opiate use, which can induce obstructive sleep apnoea, which is more liable due to the co-morbidities of obesity [23].

Applications of VR in pain management in other surgical patients have been reported to have numerous benefits. This includes a reduction in pain scores after cardiac, knee, abdominal, and spinal surgery with overall patients reporting the use of VR as a pleasant experience and stating that they would use it again on further occasions [18,24,25]. VR pain management follows a similar concept to VR and anxiety meditation by immersing patients in a simulated relaxing environment, which can help to divert the patient's feelings from their pain. This could be playing a major role in bariatric patients' management of pain and anxiety related to surgery with proper application integration in their peri-operative pathway.

Optimizing Pulmonary Function for Surgery

Respiratory function in morbidly obese patients follows a restrictive pattern with up to 77% suffering from obstructive sleep apnoea [26]. This increases the risk of impaired post-operative oxygenation and other respiratory complications in the form of atelectasis. Optimization of pulmonary function for surgery includes smoking cessation, breathing exercises, including inspiratory muscle training, incentive spirometry, and optimization of chronic disease, for example, asthma and chronic obstructive pulmonary disease (CPOD) [27].

With the increase of applications of VR in different rehabilitation programmes, VR has been aiding in pulmonary exercises in both healthy individuals and COPD patients [28,29]. VR pulmonary rehabilitation is designed to enable home-based exercises in the form of a 3D avatar instructor in an immersive relaxing environment to guide patients through breathing exercises based on traditional rehabilitation programmes [30]. In COPD patients, VR-based respiratory rehabilitation has shown to have similar outcomes when compared to a conventional programme with the additional benefit of performing the exercises from home. Moreover, VR showed enrichment of experience by also decreasing the levels of anxiety during exercise and therefore optimizing cardiorespiratory function [31].

Physical Fitness Applications

Pre- and post-operative physical activity (PA) is regarded as an important element in enhancing recovery after surgery as it improves physical state, responses to stress from surgery, and improvement of cardiovascular function, thereby reducing complications [32].

In the bariatric population, a structured exercise regime is considered a feasible and effective adjunct therapy that benefits cardiometabolic parameters when compared to those with bariatric surgery alone [33]. Exercise before surgery has shown to be beneficial in reducing body weight, improving blood pressure, general fitness, quality of life satisfaction, and decreasing fasting plasma insulin and blood lipid. Exercise after bariatric surgery has been shown to preserve dynamic muscle strength and contribute to maintaining weight loss after calorie restriction [34].

Although PA promotion is recognized as an important component of weight loss programmes, there are no current evidence-based or standardized bariatric surgery-specific PA guidelines [35]. Reported exercise regimes ranged from walking, aquatic, resistance, and supervised exercises. Also, adherence to exercise before and after surgery plays a big role in physical rehabilitation. As in the bariatric population, many can face barriers in the form of low confidence levels in their abilities and not feeling comfortable going to the gym due to real and perceived discrimination. Therefore, many come up with the belief of not having time to participate in sports [36].

VR rehabilitation has gained much recognition from dedicated platforms like treadmills, diving, cycling simulators, and medically oriented VR rehabilitation. These studies have demonstrated increased participation of users utilizing VR exercise programmes [37]. VR rehabilitation and exercise have shown to be effective in healthy individuals and different medical rehabilitations. It was reported to be equivalent and sometimes more superior to standard physiotherapy in cerebral palsy, spinal injury, and stroke [38]. In healthy individuals, VR exercise was demonstrated to increase adherence and enjoyment with positive physiological effects during exercise [39]. It was also reported that obese children performed better on treadmills while using VR than traditional walking, as VR allowed more distraction and less discomfort [40].

VR exercises during rehabilitation can therefore potentially play a major role in pre- and post-operative PA improvement in bariatric patients. Given the feasibility and the safety of these home-based devices, it can decrease the load on healthcare services, as most of the standard pre-operative programmes are resource intensive.

Virtual Reality and Enhanced Cognitive Behavioural Therapy

Eating and depressive disorders significantly affect the bariatric population with a prevalence of 24% and 17%, respectively. Both can lead to less post-operative weight loss, weight regains, impaired general psychology, and quality of life [41]. Cognitive behavioural therapy (CBT) is recommended for patients undergoing weight loss surgery (WLS). It has been shown to improve self-monitoring and control eating behaviours with significant improvement in depression and anxiety and therefore better results [42].

Over the last decades, VR-enhanced cognitive therapy (VRCBT) has been embraced for being a novel way to deliver CBT. The technique creates an interactive 3D environment to simulate successful goal achievement. This helps patients to overcome memories of previous real-life experiences through emotionally guided virtual exposure [43]. VRCBT has shown favourable results in anxiety, phobias, social anxiety disorders, and depression [21]. Moreover, randomized trials have shown VRCBT to be superior to conventional CBT in managing eating disorders and binge eating [44,45]. This helped in weight reduction therapy and adding adherence to programmes [46].

There is a paucity of evidence of the use of VR in the overweight and morbidly obese population. Phelan et al. [47] tested the use of a VR environment on 15 overweight adults for four weeks with the main hypothesis to evaluate the effect of the simulated scenes on behavioural skills related to eating habits. Although they showed no difference in weight loss among participants, VR intervention was more preferred by patients over traditional weight loss programmes [47]. Manzoni et al. [45] tested the efficacy of an enhanced VRCBT module aimed to unlock the negative memory of the body and modify its behavioural and emotional behaviour. A total of 163 female morbidly obese inpatients were randomly assigned to three CBT-based treatments: a standard behavioural inpatient programme (SBP), SBP plus standard CBT, and SBP plus VR-enhanced CBT. The study showed that patients in the VR group had a greater probability of maintaining or improving weight loss at one-year follow-up than SBP patients and, to a lesser extent, CBT patients. On the contrary, participants who received only a behavioural programme regained on average most of the weight they had lost [45].

VRCBT can therefore be a valuable tool in managing behavioural disorders related to obesity in patients undergoing WLS. This can help in maintaining weight loss and improving well-being and quality of life.

Virtual Reality and Body Image (VRBI)

Body image disorders (BIDs) are linked to various psychological and physical sequelae of impaired functions, for instance, depression, anxiety, eating disorders, and poor quality of life [48]. Among the bariatric population, body image dissatisfaction is associated with binge eating, depression, and lower self-esteem, with one in five bariatric patients identifying appearance as their main motive for surgery [49]. Improvement in body image perception after successful surgery has been linked to a decrease in compulsive eating syndromes, reduction in body mass index (BMI), and improvement in self-esteem and intimate relationships [50].

A contrary aspect of body image after surgery includes the issue of excess skin with massive weight reduction. This has been linked to poor body satisfaction, dermatitis and skin fold irritations, and impairment in daily activities and exercise. In turn, this leads 85% of bariatric patients to seek body-contouring surgery (BCS) to elevate this problem [51].

The application of VR has been used to improve BID. This is by creating a 3D simulation of their bodies in the form of avatars through an immersive environment that reproduces situations related to their body image concerns. Through multisensory simulations, it produces an empowered feeling of ownership of one’s body, which consequently promotes a healthier body image and behaviour [52]. A recent systematic review of six studies utilizing avatars and VR in weight loss programmes showed that avatar-based interventions were effective in both short- and mid-term weight loss. Also, the technology helped to improve exercise adherence in the long term [53]. VR was also used to assess the BID of 78 women with different BMIs by exposing the participants to different versions of avatars: slimmer, same weight, and overweight. The study showed that women with higher BMI reported more BID on their replicated avatar and showed satisfaction with their slimmer version. This finding indicated that VR may serve as a novel tool for measuring BID [54].

Potentially, VR avatars can also play a role in body image perception in bariatric patients. It can be integrated to improve BIDs by recreating slimmer avatars, which could promote adherence to weight loss and exercise programmes.

Smoking and Alcohol

While the increase in BMI is a risk factor for adverse outcomes related to surgical procedures, smoking's hazardous effects range from increased risks of pulmonary complications, wound infection, venous thromboembolism, and slower recovery. Similarly, alcohol consumption before surgery can lead to increased unfavourable outcomes [55]. Smoking and other substance abuse are recommended to be stopped four to six weeks pre-operatively [56]. VR has been tested as a potential solution to stop smoking and alcohol usage by inducing an advanced cue exposure therapy (CET), which was superior to static images or videos used in conventical settings [57]. Also, VR exposure therapy (VRET) has been reported to be more effective if combined with conventional cognitive behaviour therapy in relation to stopping smoking [58].

Although its applications are still under development and validation, VRET in smoking and alcohol cessation could play an important role in optimizing patients undergoing bariatric surgery as a part of a virtual reality surgical care package (VRSCP).

Discussion

Patients who are candidates for WLS usually undergo variable preparatory phase and post-operative optimization to improve both short- and long-term results. Standard care models usually involve education and follow-up through multidisciplinary teams with reflection on the patient's progress through educational sessions and follow-up plans.

While VR applications are being investigated in many surgical and medical specialities, their application to patients undergoing WLS is limited and not yet explored. The favourable applications of VR in patient education, anxiety and pain management, preoperative optimization, and behavioural and physiological treatment can be packaged as a surgical care bundle making bariatric patients' journey more satisfactory with the potential for improved outcomes.

Despite its promising applications, VR is still an emerging technology and has its own initial drawbacks to gaining traction in the healthcare system. There are several reasons for this. Firstly, the obvious cost of the systems and the absence of adequate clinical validation could play a major role in limiting widespread adoption. Further delays in adoption would likely be seen within the education of both healthcare providers and their patients, particularly on the application and utilization of the systems. The technology is still seen to be clumsy to wear and will need educational support to use [59].

With the increased investments and advancement in VR technology, education of healthcare professionals and further studies demonstrating evidence of improved outcomes, VR will play a major role in surgical patients and more specifically bariatric patients. This could be even refined as a personalized surgical care package. This will contribute to a fully virtual ecosystem in health care.

## Conclusions

VR as a new technology in healthcare services will play an increasingly important role in improving patients’ journey through surgery. VR has shown primary favourable results in managing obesity-related problems, anxiety, and pain related to surgery, improving physical fitness, physiotherapy, and other services; however, it has not yet been directed toward a patient’s package in the bariatric population. It can be utilized as a virtual ecosystem in the pre-operative stage, patient education, physical preparation, and mental health support. VR applications can be also evident in admission and peri-operative period, anxiety, and pain management. Moreover, VR applications can be ultimately useful in the post-operative phase and long-term management of bariatric patients through integration with cognitive therapy, body image disorders, and ongoing motivational fitness programmes.

Although VR is still in the early development phases, the primes of its integration into bariatric patient pathways can be limitless. Therefore, further studies into the application of VR in these patients as a surgical package (VRSCP) should contribute to a fully patient-centred virtual ecosystem for bariatric patients in the future.
